# ICTV Virus Taxonomy Profile: *Baculoviridae*

**DOI:** 10.1099/jgv.0.001107

**Published:** 2018-06-27

**Authors:** Robert L. Harrison, Elisabeth A. Herniou, Johannes A. Jehle, David A. Theilmann, John P. Burand, James J. Becnel, Peter J. Krell, Monique M. van Oers, Joseph D. Mowery, Gary R. Bauchan, ICTV Report Consortium

**Affiliations:** ^1^​ Invasive Insect Biocontrol and Behavior Laboratory, Beltsville Agricultural Research Center, USDA Agricultural Research Service, Beltsville, MD 20705, USA; ^2^​ Institut de Recherche sur la Biologie de l’Insecte, CNRS UMR 7261, Université François Rabelais, Tours 37200, France; ^3^​ Julius Kühn Institute, Federal Research Centre for Cultivated Plants, Institute for Biological Control, Darmstadt 64287, Germany; ^4^​ Summerland Research and Development Centre, Agriculture and Agri-Food Canada, Summerland, BC V0H 1Z0, Canada; ^5^​ Department of Microbiology, University of Massachusetts-Amherst, Amherst, MA 01003, USA; ^6^​ Center for Medical, Agricultural and Veterinary Entomology, USDA Agricultural Research Service, Gainesville, FL 32608, USA; ^7^​ Department of Molecular and Cellular Biology, University of Guelph, Guelph, Ontario N1G 2W1, Canada; ^8^​ Laboratory of Virology, Wageningen University, Wageningen 6709 PD, The Netherlands; ^9^​ Electron and Confocal Microscopy Unit, Beltsville Agricultural Research Center, USDA Agricultural Research Service, Beltsville, MD 20705, USA

**Keywords:** *Baculoviridae*, ICTV Report, Taxonomy

## Abstract

The family *Baculoviridae* comprises large viruses with circular dsDNA genomes ranging from 80 to 180 kbp. The virions consist of enveloped, rod-shaped nucleocapsids and are embedded in distinctive occlusion bodies measuring 0.15–5 µm. The occlusion bodies consist of a matrix composed of a single viral protein expressed at high levels during infection. Members of this family infect exclusively larvae of the insect orders Lepidoptera, Hymenoptera and Diptera. This is a summary of the International Committee on Taxonomy of Viruses (ICTV) Report on the taxonomy of the *Baculoviridae*, which is available at www.ictv.global/report/baculoviridae.

## Abbreviations

BV, budded virus; OB, occlusion body.

## Virion

**Fig. 1.**
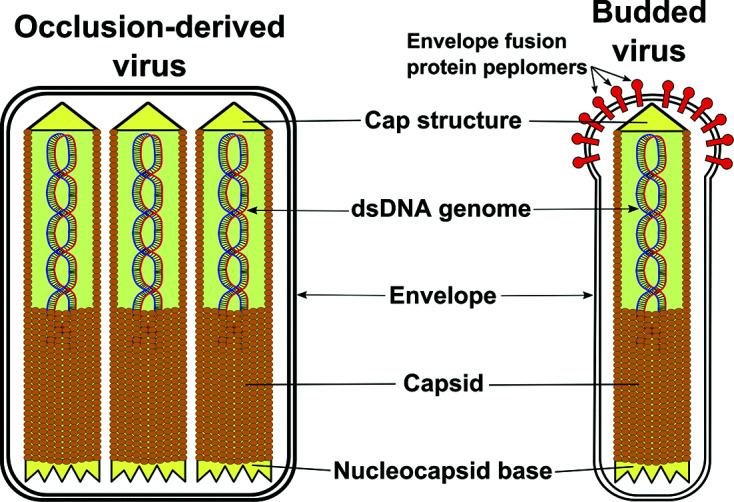
Diagram of the two baculovirus virion phenotypes.

Virions comprise cylindrical nucleocapsids, 30–60 nm in diameter × 250–300 nm within a lipid envelope (Table 1, Fig. 1). The virions, referred to as occlusion-derived virus, are embedded in occlusion bodies (OBs) comprised of a virus matrix protein. OBs usually occur as irregular polyhedra and measure 0.15–5 µm. In some genera, a second distinct type of extracellular, non-occluded virion (budded virus, BV) is also produced.

**Table 1.** Characteristics of the family *Baculoviridae*

Typical member:	Autographa californica multiple nucleopolyhedrovirus C6 (L22858), species *Autographa californica multiple nucleopolyhedrovirus*, genus *Alphabaculovirus*
Virion	One or two distinct types of virions consisting of enveloped, rod-shaped nucleocapsids, 30–60×250–300 nm, containing >20 proteins
Genome	A single covalently closed circular dsDNA molecule of 80–180 kbp encoding 100–200 proteins
Replication	Nuclear, with nucleocapsids assembled in the nucleus and enveloped either (a) in the nucleus or mixed nucleoplasm and cytoplasm, or (b) upon budding through the plasma membrane
Translation	From mRNAs transcribed from viral DNA
Host range	Larval-stage insects of orders Diptera, Hymenoptera and Lepidoptera
Taxonomy	Four genera with >60 species

## Genome

The virus genome is a single covalently-closed circular molecule of double-stranded DNA of 80–180 kbp [1] with 100 to 200 potential protein-encoding open reading frames (ORFs) that are closely spaced and occur in either orientation. ORF content and order can vary significantly between species. Most genomes also contain regions of short repeats. Thirty-eight ORFs are conserved core genes present in the genomes of all members of the family [2, 3].

## Replication

The viral genome is uncoated in the nucleus of host insect cells, and a subset of genes is transcribed by host RNA polymerase II during the early phase of replication. Progeny genomes are synthesized and assembled by a set of virally encoded proteins that include a DNA polymerase. Viral DNA synthesis marks the onset of the late phase of infection, with transcription of a separate set of genes encoding structural proteins. Nucleocapsids containing genomic DNA are assembled and enveloped within the nucleus, or in a mixed nucleo-cytoplasmic milieu resulting from disintegration of the host nuclear envelope. Mature virions are occluded in a matrix consisting of a late-phase viral protein called polyhedrin or granulin, which is synthesized at very high levels. The resulting OBs are usually released from the host after death. OBs confer a degree of environmental stability to the occluded virions and transmit infection among individual hosts. In some genera, BVs are formed when nucleocapsids acquire an envelope from the host plasma membrane while budding from the cell. BVs transmit infection to different tissues in the host.

## Taxonomy

**Fig. 2.**
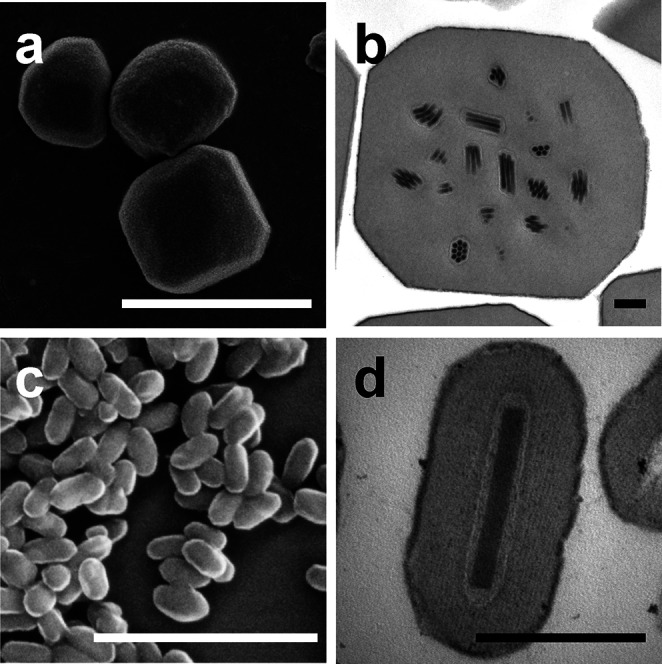
Scanning and transmission electron micrographs of occlusion bodies of (a) Operophtera brumata nucleopolyhedrovirus MA, (b) Autographa californica multiple nucleopolyhedrovirus C6, (c) Spodoptera frugiperda granulovirus and (d) Mythimna unipuncta granulovirus #8. Scale bars, (a, c) 2 µm, (b, d) 250 nm.

Members of the genus *Alphabaculovirus* infect larvae of the insect order Lepidoptera. OBs contain multiple virions with single or multiple nucleocapsids per envelope (Fig. 2a, b).

Viruses of the genus *Betabaculovirus* have been isolated exclusively from lepidopteran larvae. OBs of this genus, also known as granules, are ovocylindrical and measure approximately 0.12×0.50 µm [4]. The virions consist of a single enveloped nucleocapsid (Fig. 2c, d).

Viruses of the genus *Gammabaculovirus* replicate in the midgut of larvae of sawflies (order Hymenoptera) [5]. OBs containing virions with a single nucleocapsid are excreted from infected insects.

Viruses of the genus *Deltabaculovirus* infect the midgut of larvae of mosquitoes (order Diptera) [6] and encode a matrix protein unrelated to those of other baculoviruses.

## Resources

Full ICTV Online (10th) Report: www.ictv.global/report/baculoviridae.

Baculovirus Molecular Biology, 3rd edition: https://www.ncbi.nlm.nih.gov/books/NBK114593/.
